# Siglec-5 is a novel marker of critical limb ischemia in patients with diabetes

**DOI:** 10.1038/s41598-017-11820-x

**Published:** 2017-09-12

**Authors:** Ju-yi Li, Xiao-yan Yang, Xiu-fang Wang, Xiong Jia, Zhong-jing Wang, Ai-ping Deng, Xiang-li Bai, Lin Zhu, Bing-hui Li, Zi-bo Feng, Ye Li, Ling Wang, Si Jin

**Affiliations:** 1 Department of Pharmacology, Hubei Key Laboratory of Drug Target Research and Pharmacodynamic Evaluation, School of Basic Medicine, Tongji Medical College, Huazhong University of Science and Technology, Wuhan, Hubei China; 2 Department of Endocrinology, Institute of Geriatric Medicine, Liyuan Hospital, Tongji Medical College, Huazhong University of Science and Technology, Wuhan, Hubei China; 3 Department of Pharmacy, The central Hospital of Wuhan, Tongji Medical College, Huazhong University of Science and Technology, Wuhan, Hubei China; 4 Department of Pain, The central Hospital of Wuhan, Tongji Medical College, Huazhong University of Science and Technology, Wuhan, Hubei China; 5 Department of Endocrinology, The central Hospital of Wuhan, Tongji Medical College, Huazhong University of Science and Technology, Wuhan, Hubei China; 6 Department of Wound Repair, Liyuan Hospital, Tongji Medical College, Huazhong University of Science and Technology, Wuhan, Hubei China

## Abstract

Critical Limb Ischemia (CLI) is common but uncommonly diagnosed. Improved recognition and early diagnostic markers for CLI are needed. Therefore, the aim of our study was to identify plasma biomarkers of CLI in patients with type 2 diabetes mellitus (T2DM). In this study, antibody-coated glass slide arrays were used to determine the plasma levels of 274 human cytokines in four matched cases of diabetes with and without CLI. Potential biomarkers were confirmed in an independent cohort by ELISA. After adjusting for confounding risk factors, only plasma level of Siglec-5 remained significantly associated with an increased odds ratio (OR) for diabetes with CLI by binary logistic regression analysis. Receiver operating characteristic (ROC) curve analysis revealed the optimal cut-off points for Siglec-5 was 153.1 ng/ml. After entering Siglec-5, the AUC was 0.99, which was higher than that of confounding risk factors only (AUC = 0.97, *P* < 0.05). Siglec-5 was expressed in plaques, but not in healthy artery wall in T2DM patients. Elevated plasma Siglec-5 was independently associated with CLI in T2DM. Plasma Siglec-5 levels are implicated as an early diagnostic marker of CLI in T2DM patients and it may become a target for the prevention or treatment of CLI in diabetes.

## Introduction

Type 2 diabetes mellitus (T2DM) is associated with accelerated atherosclerosis and a 2‒3-fold increased risk of acute myocardial infarction, stroke and peripheral arterial disease (PAD)^1^. PAD and infection are the major causes of delayed healing of foot wounds and lower leg amputation in patients with diabetes^2^. Critical limb ischemia (CLI) caused by PAD is the result of seriously decreased blood flow to the extremities, which increases the risk of foot ulceration, amputation and vascular death. This condition seriously reduces functional capacity and quality of life^3–5^. However, little information is available regarding CLI, particularly among subjects with T2DM.

T2DM is a major contributor to CLI. The biological mechanisms by which T2DM causes changes in lower limb vasculature remain to be fully elucidated, but are believed to involve oxidative and glycemic stress, chronic low grade inflammation, and impaired vascular tissue repair^6–8^. The goal of CLI therapy is to reduce cardiovascular risk factors, morbidity and mortality through statins and antiplatelet therapy, revascularization, amputation^9, 10^. Currently, several tests are applied to confirm the diagnosis of CLI in diabetes patients, such as the ankle-brachial index (ABI), color Doppler ultrasound examination, and transcutaneous oxygen pressure^11^. Unfortunately, CLI is underdiagnosed and undertreated because many patients with PAD do not manifest classic symptomatology. Only 10% to 30% of patients with PAD have the classic clinical symptoms of intermittent claudication, and almost half of patients are long-term sedentary leading to an absence of symptoms^12^. As a result, patients without clinical symptoms did not receive active treatment. Accordingly, blood tests would be a useful and convenient method for identification of a predictor or diagnostic marker of the disease.

Indeed, specific serum or plasma proteins including Cystatin C, haptoglobin, 14-3-3 protein zeta, and leucine-rich α-2-glycoprotein have been reported as potential biomarkers of CLI in diabetes^4, 5^, however, development of these candidate biomarkers from bench-to-bedside is a lengthy process. Clearly, early recognition of CLI in T2DM patients, an accurate estimation of its severity and timely and effective application of measures for prevention and intervention are of great significance in reducing the high number of amputations. Therefore, more valuable biomarkers are urgently required as early predictors or diagnostic markers of CLI in T2DM patients.

High-throughput antibody arrays are designed for the detection of specific antigens or antibodies and allow screening of large numbers of protein biomarker candidates in cell culture media, tissues or body fluids. This technique is widely used in various fields including cancer, autoimmune diseases, and diabetes^13–15^. In the present study, a discovery study was conducted in plasma samples of T2DM patients (4 with CLI and 4 matched patients without CLI) using an antibody array containing 274 potential biomarker proteins (including chemokines and mediators of processes such as inflammation, angiogenesis and atherosclerosis). Based on these screening tests, 11 proteins were selected for confirmation in an independent cohort using ELISA assays. Our result suggests that elevated Siglec-5 levels are an independent predictor of CLI in patients with T2DM.

## Results

### Patient characteristics

Details of the patient characteristics are shown in Table 1. The initial biomarker screen of plasma from T2DM patients with and without CLI (*n* = 4/group) was performed using protein arrays. There were significant differences between the groups in ABI and SBP (both *P* < 0.05), but no differences in terms of age, sex, T2DM duration, smoking or drinking history, BMI, CAD history, fasting plasma glucose (FPG) and HbA1c (all *P* > 0.05). In the confirmation study, samples from non-CLI (*n* = 108) and CLI subjects (*n* = 59) were analyzed by ELISA. There were significant differences between the groups in age, T2DM duration, sex, hypertension, ABI, SBP, TG, TC, Apo A, Cr, BUN, and total bilirubin (all *P* < 0.05), but no differences in hyperlipidemia, smoking or drinking history, BMI, CAD history, FPG or HbA1c (all *P* > 0.05).

**Table 1:** Demographics and Clinical Characteristics of Diabetes Patients.

	protein array cohort		*P*	confirmation cohort		*P*
	CLI	Non - CLI		CLI	Non - CLI	
No. of subjects	4	4	–	59	108	–
Age, y	59.50 ± 6.44	61.25 ± 6.98	0.831	72.07 ± 1.52	53.97 ± 1.02	*0*.*000*
T2D Duration, y	7.75 ± 1.55	10.50 ± 2.26	0.458	12.91 ± 1.05	5.1 (2.1–8.8)	*0*.*000* ^†^
Women, %	2,(50.00)	2,(50.00)	1.000	22,(37.29)	59,(54.63)	*0*.*032* ^‡^
Hyperlipidemia, %	2,(50.00)	1,(25.00)	1.000	34,(57.63)	76,(70.37)	0.097^‡^
Hypertension, %	3,(75.00)	1,(25.00)	0.486	45,(76.27)	54,(50.00)	*0*.*001* ^‡^
Drinking, %	3,(75.00)	0,(0)	0.143	10,(16.95)	18,(16.67)	0.963^‡^
Smoking, %	3,(75.00)	1,(25.00)	0.486	19,(32.20)	28,(25.93)	0.389^‡^
ABI	0.84 ± 0.00	1.07 ± 0.03	*0*.*030*	0.84 ± 0.03	1.11 ± 0.02	*0*.*000*
BMI, kg/m^2^	25.28 ± 1.73	23.94 ± 1.34	0.528	25.21 ± 0.41	24.83 ± 0.28	0.456
CAD, %	3,(75.00)	1,(25.00)	0.486	24,(40.68)	29,(26.85)	0.067^‡^
Statin use, %	2,(50.00)	1,(25.00)	1.000	25,(42.37)	33,(30.56)	0.125^‡^
Antihypertensive treatment use, %	3,(75.00)	1,(25.00)	0.486	27,(45.76)	35,(32.41)	0.088^‡^
SBP, mm Hg	143.75 ± 4.73	116.75 ± 3.61	*0*.*031*	142.25 ± 3.39	120 (115–135)	*0*.*000* ^†^
DBP, mm Hg	76.25 ± 2.39	70.50 ± 1.85	0.204	77.37 ± 1.34	75 (70–83)	0.594^†^
FPG, mmol/L	11.50 ± 1.87	8.81 ± 2.12	0.380	11.55 ± 1.01	8.14 (6.38–11.87)	0.082^†^
HbA1c, %	10.35 ± 1.15	8.33 ± 1.09	0.247	8.83 ± 0.30	8.20 (6.40–9.50)	0.103^†^
HDL-C, mmol/L	0.95 ± 0.08	1.06 ± 0.13	0.475	1.04 ± 0.05	1.13 ± 0.03	0.156
LDL-C, mmol/L	3.36 ± 0.82	2.78 ± 0.42	0.661	2.44 ± 0.13	2.71 (1.90–3.28)	0.075^†^
TG, mmol/L	1.10 ± 0.14	1.26 ± 0.25	0.614	1.21 (0.83–1.79)	1.63 (1.01–2.34)	*0*.*032* ^†^
TC, mmol/L	4.82 ± 0.97	4.23 ± 0.56	0.648	4.06 ± 0.14	4.51 ± 0.10	*0*.*011*
Apo A, g/L	1.11 ± 0.09	1.15 ± 0.05	0.798	1.11 ± 0.03	1.24 ± 0.02	*0*.*001*
Apo B, g/L	1.00 ± 0.16	0.84 ± 0.09	0.545	0.87 ± 0.03	0.90 ± 0.02	0.495
Cr, μmol/L	56.73 ± 4.72	62.00 ± 2.48	0.398	72.50 (60.00–86.80)	64.13 ± 2.00	*0*.*000* ^†^
BUN, mmol/L	4.75 ± 0.79	5.12 ± 0.74	0.668	5.75 (4.75–7.62)	5.18 ± 0.17	*0*.*003* ^†^
UA, mmol/L	303.85 ± 40.39	342.50 ± 25.51	0.505	323.53 ± 12.93	319.95 ± 11.14	0.840
Total bilirubin, μmol/L	14.08 ± 2.51	14.73 ± 2.31	0.798	11.13 ± 0.65	14.15 (10.77–18.83)	*0*.*000* ^†^

ABI, Ankle brachial index; BMI, body mass index; CAD, coronary artery disease; SBP, systolic blood pressure; DBP, diastolic blood pressure; FPG, fasting plasma glucose; HbA1c, glycosylated hemoglobin; HDL-C, high density lipoprotein cholesterol; LDL-C, low density lipoprotein cholesterol; TG, triglyceride; TC, total cholesterol; Apo A, Apolipoprotein A; Apo B, Apolipoprotein B; Cr, serum creatinine; BUN, blood urea nitrogen; UA, uric acid. Data is shown as mean ± SEM, median (interquartile range) or percentage (%) of subjects in each group. Significant values are marked in italic. Differences between the groups in the protein array cohort were analyzed using the Fisher-Pitman Permutation test for equality of means and using Fisher’s exact test for categorical variables, however, the confirmation cohort was analyzed using ^†^) Mann-Whitney U-test or ‡)*χ* ^2^ test, all the others were analyzed using *t*-tests.

### High-throughput protein arrays analysis

**Figure 1:**
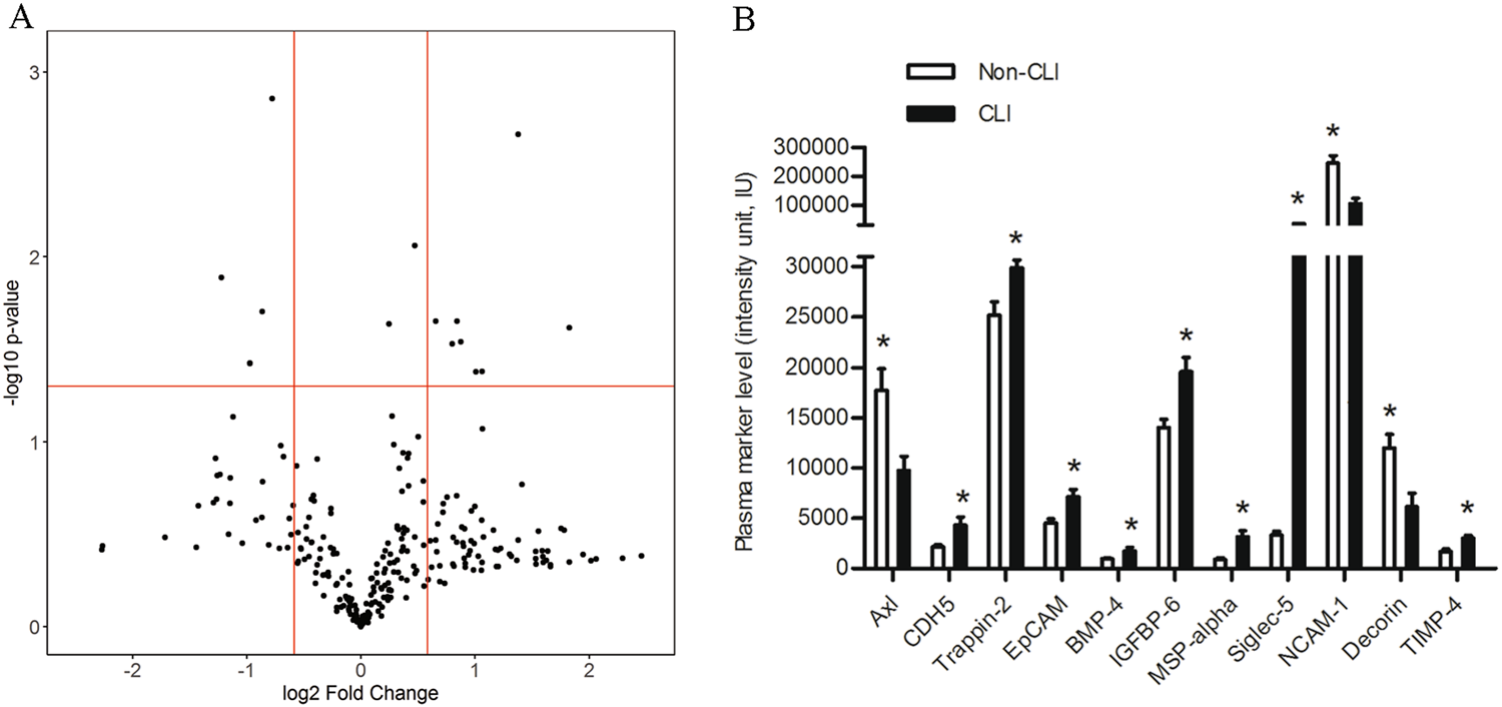
High-Throughput Protein-based screening of diabetes with CLI for biomarkers. Plasma samples levels of 274 proteins from patients with CLI (*n* = 4) and without CLI (*n* = 4) were tested using antibody arrays. (**A**) Volcano plot of the expression profiles of the 274 proteins expressed as a fold change (in CLI *vs* non-CLI control plasma) and statistical significance of the difference (both expressed in log scales). (**B**) Proteins that exhibited significant increases or decreases in CLI patients (*n* = 4) compared with non-CLI patients (*n* = 4) in the antibody array analysis, where the CLI patients and non-CLI (*n* = 4) patients are represented as black and white bars, respectively, *P < 0.05.

After multiple testing correction, eleven plasma proteins showed significantly different expression in T2DM patients with CLI compared with those without CLI. Of these, eight plasma proteins were significantly elevated (CDH5, Trappin-2, EpCAM, BMP-4, IGFBP-6, MSP-alpha, Siglec-5 and TIMP-4), while three were significantly downregulated (Axl, NCAM-1 and Decorin) (Fig. 1A,B).

### Confirmation study of biomarkers for CLI in T2DM

**Figure 2:**
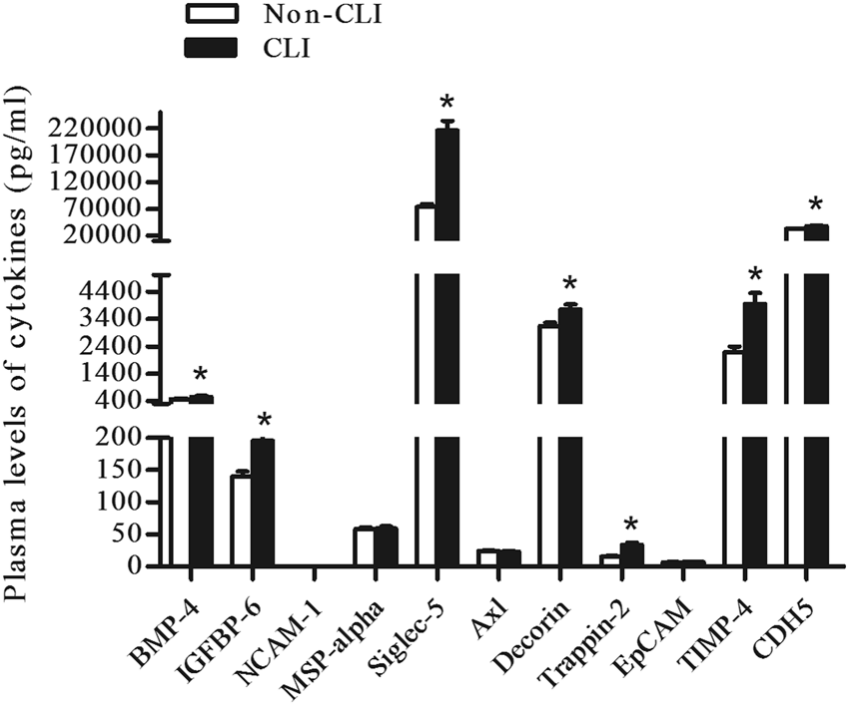
A confirmation study for the findings of antibody array analysis (11 potential proteins) in an independent cohort subjects with CLI (*n* = 59) and non-CLI (*n* = 108) patients by ELISA. The plasma levels of the 11 proteins in the respective study groups. BMP-4, IGFBP-6, Siglec-5, Decorin, Trappin-2, TIMP-4, and CDH5 were significantly elevated in CLI compared with non-CLI (^*^ *P* < 0.05).

ELISA analysis results of the eight samples tested in protein arrays yielded results were consistent with that of the protein arrays. We then performed a confirmation study in an independent cohort of T2DM patients with CLI (*n* = 59) and without CLI (*n* = 108) by ELISA. As shown in Table 2 and Fig. 2, among the 11 potential biomarkers, seven (BMP-4, IGFBP-6, Siglec-5, Decorin, Trappin-2, TIMP-4, and CDH5) were significantly elevated in the CLI group compared with the non-CLI group, while there were no significant differences in the levels of NCAM-1, MSP-alpha, Axl or EpCAM.

**Table 2:** Confirmation assays of plasma proteins in diabetes and their potential to discriminate lower limb sclerosis from controls (pg/ml).

Proteins	protein array cohort^†^		confirmation cohort	
	CLI (*n* = 4)	Non-CLI (*n* = 4)	CLI (*n* = 59)	Non-CLI (*n* = 108)
BMP-4	497.34 ± 26.24^#^	385.74 ± 20.66	558.57 ± 34.20^#^	437.14 (330.11–544.50)^‡^
IGFBP-6	209.06 ± 21.49^#^	99.93 ± 17.95	195.62 ± 19.59^#^	124.14 (88.49–167.22)^‡^
NCAM-1	(182.92 ± 11.42)*10^−3#^	(277.58 ± 18.64)*10^−3^	(264.20 ± 8.25)*10^−3^	(264.40 ± 7.11)*10^−3^
MSP-alpha	57.34 ± 6.99^#^	31.60 ± 3.14	59.30 ± 3.21	58.79 ± 2.13
Siglec-5	(258.76 ± 37.21)*10^3#^	(45.66 ± 6.00)*10^3^	(193.62 (158.08–196.32))*10^3###^	(73.84 ± 4.76)*10^3‡^
Axl	14.64 ± 1.48^#^	26.71 ± 1.64	23.33 ± 1.36	21.37 (13.74–29.02)^‡^
Decorin	(1.90 ± 0.30)*10^3#^	(3.52 ± 0.16)*10^3^	(3.75 ± 0.18)*10^3##^	(3.14 ± 0.12)*10^3^
Trappin-2	33.42 ± 2.78^#^	14.08 ± 2.49	23.22 (13.23–50.34)^###^	13.22 (9.43–19.02)^‡^
EpCAM	10.23 ± 0.23^#^	5.00 ± 1.25	7.30 ± 0.68	6.50 (1.38–10.88)^‡^
TIMP-4	(4.19 ± 0.30)*10^3#^	(2.08 ± 0.21)*10^3^	(3.94 ± 0.41)*10^3###^	(2.18 ± 0.22)*10^3^
CDH5	(40.26 ± 1.77)*10^3#^	(29.47 ± 1.59)*10^3^	(37.92 ± 1.23)*10^3###^	(32.92 ± 0.56)*10^3^

Data is shown as mean ± SEM, median (interquartile range) of subjects in each group. Differences between the groups in the protein array cohort were analyzed using †) Fisher-Pitman Permutation test, however, the confirmation cohort was analyzed using ‡) Mann-Whitney U-test, all the others were analyzed using *t*-tests. ^#^ *p* < 0.05; ^##^ *p* < 0.01^; ###^ *p* < 0.001.

### Analysis of plasma cytokine levels and CLI risk factors

With the exception of BMP-4, the plasma cytokine levels increased with age, T2DM duration or BUN (all *P* < 0.05). The plasma levels of cytokines (Decorin, Siglec-5 and TIMP-4) increased with SBP (all *P* < 0.05). There were negative associations between TC and plasma Siglec-5 or TIMP-4 levels (all *P* < 0.05). There were also negative associations between Apo A and plasma Trappin-2, Siglec-5 or CDH5 levels (all *P* < 0.05). With the exception of BMP-4 and Decorin, the plasma cytokine levels increased with Cr (all *P* < 0.05). In addition, increased plasma levels of Trappin-2, IGFBP-6, Siglec-5 and TIMP-4 correlated with lower total bilirubin levels (all *P* < 0.05) (Table 3).

**Table 3:** Spearman Rho correlations analysis of plasma cytokine levels and CLI risk factors.

Variable		Trappin-2	IGFBP-6	BMP-4	Decorin	siglec-5	TIMP-4	CDH5
Age	*rho*	0.421	0.276	0.136	0.356	0.517	0.507	0.224
	*p*	*2*.*06E-8*	*3*.*44 E-4*	0.085	*4*.*75E-6*	*8*.*75E-13*	*4*.*13E-6*	*0*.*005*
T2D Duration	*rho*	0.333	0.331	0.098	0.231	0.267	0.406	0.187
	*p*	*1*.*34E-5*	*1*.*49E-5*	0.216	*0*.*004*	*4*.*76E-4*	*3*.*30E-4*	*0*.*018*
SBP	*rho*	0.072	0.054	−0.076	0.186	0.306	0.301	0.104
	*p*	0.356	0.496	0.335	*0*.*019*	*5*.*65E-5*	*0*.*009*	0.192
TC	*rho*	−0.150	−0.043	0.121	−0.006	−0.170	−0.278	−0.137
	*p*	0.064	0.595	0.137	0.940	*0*.*033*	*0*.*021*	0.097
Apo A	*rho*	−0.238	0.046	0.014	0.028	−0.186	−0.117	−0.174
	*p*	*0*.*003*	0.574	0.861	0.738	*0*.*021*	0.349	*0*.*037*
Cr	*rho*	0.520	0.497	0.015	0.075	0.214	0.422	0.187
	*P*	*3*.*36E-12*	*4*.*07E-11*	0.849	0.361	*0*.*007*	*3*.*05E-4*	*0*.*022*
BUN	*rho*	0.376	0.348	0.014	0.185	0.305	0.343	0.187
	*p*	*1*.*23E-6*	*7*.*99E-6*	0.867	*0*.*023*	*8*.*61E-5*	*0*.*004*	*0*.*021*
Total bilirubin	*rho*	−0.288	−0.208	−0.133	−0.073	−0.309	−0.317	−0.137
	*p*	*2*.*53E-4*	*0*.*009*	0.099	0.372	*7*.*07E-3*	*0*.*007*	0.093

All study subjects were included in the analysis. Significant values are marked in italic.

### Risk factors for CLI

Taking CLI as the dependent variable, the risk factors shown in Table 1 were entered into binary logistic regression analysis. The significant risk factors included sex, age, T2DM duration, SBP, TC, Apo A, Cr, BUN, total bilirubin, Trappin-2, IGFBP-6, BMP-4, Decorin, Siglec-5, TIMP-4 and CDH5 (Table 4). TG was included in our multivariable analysis and proved not to be significant (*P* = 0.053); therefore, this factor was excluded from our final model.

**Table 4:** Risk factors for CLI in diabetes by binary logistic regression analysis.

	OR	95% CI for OR	*P*	OR^*^	95% CI for OR^*^	*P* ^*^
Sex	0.494	0.258–0.946	0.033	∕	∕	∕
Age	1.160	1.110–1.214	0.000	∕	∕	∕
T2D Duration	1.163	1.098–1.231	0.000	∕	∕	∕
SBP	1.041	1.023–1.061	0.000	∕	∕	∕
TC	0.652	0.466–0.913	0.013	∕	∕	∕
Apo A	0.061	0.011–0.333	0.001	∕	∕	∕
Cr	1.024	1.008–1.039	0.002	∕	∕	∕
BUN	1.319	1.103–1.577	0.002	∕	∕	∕
Total bilirubin	0.867	0.807–0.931	0.000	∕	∕	∕
Trappin-2	1.065	1.035–1.095	0.000	0.987	0.933–1.043	0.637
IGFBP-6	1.005	1.001–1.008	0.005	0.993	0.984–1.002	0.117
BMP-4	1.001	1.000–1.003	0.039	1.001	0.997–1.004	0.709
Decorin	1.000	1.000–1.001	0.005	1.000	0.999–1.001	0.744
Siglec-5	1.043	1.029–1.057	0.000	1.077	1.028–1.128	*0*.*002*
TIMP-4	1.000	1.000–1.001	0.003	0.994	0.000–3066.573	0.999
CDH5	1.106	1.050–1.166	0.000	1.205	1.003–1.449	0.057

CI, confidence interval. Logistic regression models were used to calculate OR. ^*^Adjusted for sex, age, T2D Duration, SBP, TC, Apo A, Cr, BUN, Total bilirubin. pg/ml (Trappin-2, IGFBP-6, BMP-4), ng/ml (Decorin, Siglec-5, TIMP-4). All study subjects were included in the analysis. Significant values are marked in italic.

After adjusting for sex, age, T2DM duration, SBP, TC, Apo A, Cr, BUN and total bilirubin (the confounding risk factors and the plasma cytokines were all entered into covariates by binary logistic regression analysis), plasma levels of Siglec-5 remained a significant association with an increased odds ratio (OR) for diabetes with CLI (*P* < 0.05), whereas BMP-4, Decorin and CDH5 were associated with an increased OR for diabetes with CLI, and Trappin-2, IGFBP-6 and TIMP-4 were associated with a lower OR for CLI, although this did not reach the level of statistical significance in this study cohort (Table 4).

### Siglec-5 expression in human lower extremity atherosclerotic plaques

**Figure 3:**
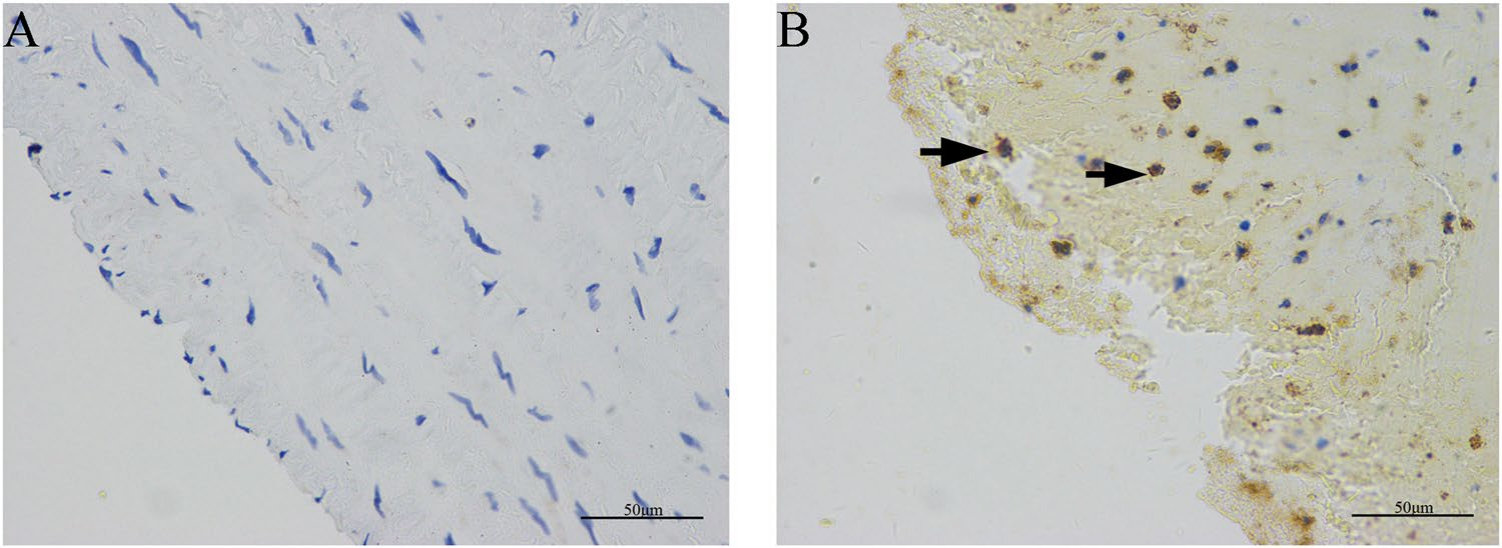
Expression of Siglec-5 in human lower extremity atherosclerotic lesions. In T2DM patients without PAD, the normal lower extremity artery tested negative for Siglec-5 staining (**A**). In contrast, Siglec-5 was detected in atherosclerotic plaques in T2DM patients (**B**). Arrows represent examples of positive staining. Scale bar = 50 μm.

We performed immunohistochemistry staining for Siglec-5 in human atherosclerotic plaques of lower extremity arteries in comparison to healthy artery wall in T2DM patients. As shown in Fig. 3B, atherosclerotic plaques expressed high levels of Siglec-5. In contrast, healthy artery walls were negative for siglec-5 (Fig. 3A).

### Diagnostic value of Siglec-5 for CLI

**Figure 4:**
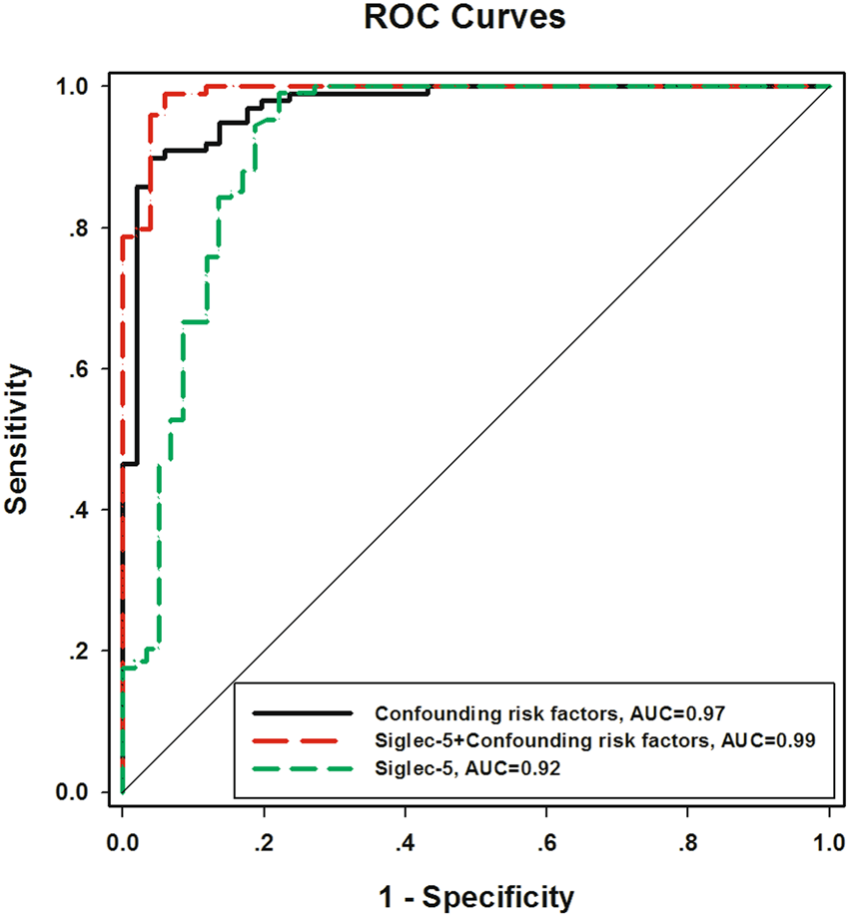
Analysis of ROC curve to detect CLI in diabetes patients. In ROC analysis, the AUC of Siglec-5 was 0.92, the AUC of confounding risk factors was 0.97 and the AUC of Siglec-5 + Confounding risk factors was 0.99.

ROC curve analysis was performed to verify the diagnostic accuracy of Siglec-5 for CLI in T2DM based on the data of all study subjects including the “protein array cohort” and the “confirmation study cohort”. The area under the curve (AUC) of Siglec-5 was 0.92 (95% confidence intervals (CI) 0.864–0.971, *P* < 0.001) and the optimal cut-off point for Siglec-5 was 153.1 ng/mL, which could be used as a diagnostic cut-off point in plasma for CLI in T2DM patients. At this level, the Youden index = 0.77, sensitivity was 99.07% (95% CI 0.949–0.999); specificity was 77.97% (95% CI 0.652–0.877). The AUC of Siglec-5 + Confounding risk factors was 0.99 (95% CI 0.979–1.002, *P* < 0.001), which was higher than that of confounding risk factors (AUC = 0.97, 95% CI 0.948–0.997, *P* < 0.001) and Siglec-5 (*P* < 0.05 and *P* < 0.01, respectively) (Fig. 4).

## Discussion

In the present study, plasma levels of Siglec-5 were found to be significantly elevated in T2DM patients with CLI compared with those without CLI, even after adjusting for confounding risk factors (sex, age, T2DM duration, SBP, TC, Apo A, Cr, BUN and total bilirubin). Siglec-5 was independently and strongly associated with T2DM patients with CLI. A previous study of differentially expressed plasma proteins between hemodialytic diabetic patients with and without CLI was performed using Matrix-Assisted Laser Desorption/Ionization Time of Flight Mass spectrometry^5^, however, the subjects were all dialysis patients, a population that was not included in our study. Therefore, the present study is the first to employ high-throughput antibody arrays to identify biomarkers of CLI in T2DM. Our findings implicate Siglec-5 level as an early diagnostic marker of T2DM patients with CLI (especially in T2DM patients without overt cerebrovascular disease).

CLI caused by arteriosclerosis, plaque formation and arterial occlusions is one of the manifestations of PAD. In the T2DM population, sex, age, smoking status, T2DM duration, hyperlipidemia, hypertension and blood glucose are the traditional risk factors for CLI^16^. Similarly, we found significant differences in sex, age, T2DM duration and hypertension between T2DM patients with and without CLI in our study. However, TG, TC and Apo A were lower in patients with CLI (all *P* < 0.05), possibly due to the diversity of the study population and the poor nutritional status in patients with CLI. In addition, we also found that T2DM patients with CLI had higher plasma levels of Cr and BUN, but lower plasma level of total bilirubin, which is consistent with previous studies^4, 17^.

Conformation by ELISA study in a cohort revealed that seven (CDH5, Trappin-2, BMP-4, IGFBP-6, Siglec-5, TIMP-4 and Decorin) of the 11 plasma proteins were significantly elevated (1.1–2.9-fold) in T2DM patients with CLI compared to those without CLI. Correlation analysis showed that the levels of the vast majority of these plasma proteins increased with age, T2DM duration, Cr and BUN. However, some plasma proteins correlated with lower levels of Apo A, TC and total bilirubin. Thereafter, we confirmed the relationship of Siglec-5 with CLI in T2DM using binary logistic regression analysis to control the confounding risk factors (sex, age, T2DM duration, SBP, TC, Apo A, Cr, BUN and total bilirubin). This analysis revealed that Siglec-5 was independently and strongly associated with prevalent T2DM patients with CLI. Immunohistochemical staining of the sections prepared from atherosclerotic plaques revealed Siglec-5 expression, while healthy artery walls in T2DM patients were Siglec-5 negative. This evidence further confirmed a relationship between Siglec-5 and CLI in T2DM patients. Finally, ROC curve analysis showed that the AUC of Siglec-5 + Confounding risk factors was higher than that of confounding risk factors (*P* < 0.05), which provided further evidence confirming that Siglec-5 plays an important role in the prediction model for CLI in T2DM patients.

The Siglec family of proteins are sialic-acid-binding immunoglobulin-like lectins which promote cell-cell interactions and regulate the functions of cells in the innate and adaptive immune systems through glycan recognition^18, 19^. The first Siglec to be characterized was the macrophage receptor sialoadhesin/Siglec-1^20^. Research on the relationship between Siglecs and atherosclerosis has focused mainly on Sigelc-1^21–23^. Siglec-5 is a member of the CD33-related Siglec subfamily, with four extracellular Ig-like domains and two intra-cellular tyrosine-based signaling motifs^24^, and is found on monocytes and neutrophils as well as on macrophages^25, 26^. Siglec-5 is involved in the regulation of innate immune responses^14, 27–29^. Although, innate and adaptive immune responses play important roles in the pathogenesis of atherosclerosis^30^, the relationship between Siglec-5 and atherosclerosis remains to be established. In this study, we identified a significant association between Siglec-5 and CLI in T2DM patients even after adjustment for confounding risk factors. More interestingly, Siglec-5 can clearly be observed in atherosclerotic plaques, but not in healthy controls in T2DM patients. This is the first study to demonstrate a strong relationship between the plasma levels of siglec-5 and CLI in T2DM patients. Monocytes can adhere to vascular endothelial cells through the interaction of Siglec-5 with P-selectin glycoprotein ligand-1 or von Willebrand factor^27, 31^, and are recruited to the blood vessel wall by deformation and chemotaxis, resulting in transformation into macrophages. Ox-LDL tends to bind to glycosylated agglutinin-like receptors and Siglec-5 glycosylation may be increased in diabetic environment, thereby facilitating the combination of Siglec-5 with Ox-LDL^32, 33^. This interaction causes macrophages to develop into lipid-rich foam cells, thereby promoting the formation of atherosclerotic plaques. Although the immune response plays an important role in the pathogenesis of atherosclerosis, the relationship between Siglec-5 and atherosclerosis remains to be established.

CLI is underdiagnosed and undertreated when traditional detection methods are used for confirmation. CLI in T2DM patients has several important characteristics that render it difficult to treat. Clearly, early recognition of CLI in T2DM patients will be beneficial in the application of timely and effective measures for prevention and or treatment which may reduce the high number of amputations. Therefore, it is especially important to identify valuable biomarkers for early diagnostic markers of T2DM patients with CLI. There are some limitations of the present study that should be considered. Although this study involved high-throughput antibody arrays of 274 potential biomarker proteins (including chemokines, inflammatory, angiogenesis, atherosclerosis and other mediators), this panel is not exhaustive. It is estimated that there are more than 10 000 proteins in human plasma, thus, in the present study, we analyzed fewer than 3% of all the proteins and further studies are required to investigate a more comprehensive panel of plasma proteins. Although this cross-sectional study revealed that Siglec-5 is associated with CLI in T2DM patients, the relationship between clinical outcomes in T2DM patients with CLI and elevated Siglec-5 levels in plasma and the precise molecular mechanism underlying the influence of Siglec-5 on the development of atherosclerosis remains to be determined in further prospective studies.

In conclusion, the present findings demonstrate that elevated plasma Siglec-5 is independently associated with T2DM and CLI, and with an increased incidence of CLI in T2DM. Plasma Siglec-5 levels are implicated as an early diagnostic marker of CLI in T2DM patients with the potential for development of strategies for the prevention or treatment of diabetes with CLI.

## Materials and Methods

### Subjects

This study was approved by the Ethics Committee of the Central Hospital of Wuhan and was conducted in accordance with the principles of the Declaration of Helsinki. All samples were collected with written informed consent provided by the participants. A total of 839 patients with T2DM (including patients with CLI who underwent peripheral cutting balloon angioplasty and patients without PAD who underwent amputation due to trauma) were recruited from the Central Hospital of Wuhan from May 2015 to September 2016. T2DM was diagnosed according to the WHO 1999 criteria and 2012 American Diabetes Association standards^34^.

CLI defined as ankle-brachial index (ABI) <0.9 and lower extremity arterial stenosis >50% was determined by digital subtraction angiography, computed tomography angiography or color Doppler ultrasound examination. Non-CLI was defined as normal lower extremity arterial pressure and with 0.9 ≤ ABI < 1.3. Non-CLI controls were appropriately matched for sex, age (± 5 years) and diabetes duration (±5 years) to the studied group of patients with CLI in the initial discovery study.

According to these criteria, 175 patients from this cohort (*n* = 839) were recruited into this study and divided into two groups: subjects with T2DM and CLI (*n* = 63) and subjects with T2DM and without CLI (*n* = 112). In total, 90 males (51.4%) and 85 females (48.6%), with an average age of 60 ± 14 years and diabetes duration of 9 ± 7 years, were selected for inclusion in this study.

Exclusion criteria were: Patients with type 1 diabetes, diagnosed with cancer, severe liver and kidney failure, on therapy for any chronic inflammatory disease.

### Information collection

The following clinical information and biochemical characteristics of all patients were collected: sex, age, diabetes duration, body mass index (BMI), blood pressure, smoking and drinking status, blood glucose, glycosylated hemoglobin (HbA1c), white blood cell count, lipoprotein lipid levels and history of hypertension, hyperlipidemia, and coronary artery disease (CAD). Hypertension was defined as diastolic blood pressure ≥90 mmHg, systolic blood pressure ≥140 mmHg or history of anti-hypertensive medication. Hyperlipidemia was defined as total cholesterol ≥5.72 mmol/L, triglyceride ≥1.70 mmol/L or use of lipid-lowering drugs. CAD was defined as a history of myocardial infarction or angina or coronary arterial stenosis > 50%.

### Targeted Protein Array

Plasma samples were obtained from T2DM patients with CLI (*n* = 4) and T2DM diabetes without CLI controls (*n* = 4) matched for age, sex, diabetes duration and ethnicity. Equal amounts of protein were loaded in all samples, which were diluted 2-fold in sample buffer (1% BSA in PBS), and plasma levels of 274 different human proteins were analyzed using the RayBio Human Cytokine Antibody Array G - Series 4000 (cat. no. AAH-CYT-G4000-8) glass slide arrays (Raybiotech, Norcross, Inc., GA, USA). Duplicate assays were designed for each protein in the slides. The slides were then scanned using an InnoScan 300 Microarray Scanner (Innopsys, Carbonne, France). Raw fluorescence data were processed using Q-Analyzer software (RayBiotech, Inc., Norcross, GA, USA). Individual spot fluorescence data were normalized using the fluorescence intensities of background and positive controls.

### ELISA Assay

Plasma samples were stored at −80 °C. Potential biomarkers were confirmed using ELISA kits (Raybiotech, Norcross, GA, USA) according to the manufacturer’s instructions. The optical density of each well was determined using a microplate reader at 450 nm. For each assay, plasma was diluted 1: 1‒1: 200 into sample diluents, and duplicate assays were performed for each sample.

### Immunohistochemistry

Immunohistochemistry was performed on representative sections (5 μm) of formalin-fixed in paraffin-embedded tissue from human lower extremity atherosclerotic lesions (*n* = 3) and healthy artery wall controls in diabetes (amputation due to trauma, *n* = 3). For detection of Siglec-5, the sections were stained using anti-human Siglec-5 rabbit polyclonal antibody (13230-1-AP, Proteintech Group) diluted 1:100 dilution. Sections were incubated at 4 °C, and then incubated with HRP-conjugated secondary antibodies according to the immunostaining procedure.

### Statistics

Data were plotted and analyzed using SPSS 19.0 (SPSS Inc., Chicago, IL, USA) or GraphPad Prism 5 (GraphPad, San Diego, CA, USA). Data were expressed as means ± SEM, median (interquartile range) or percentages (%). Fisher’s exact test or x^2^ test was used for categorical data. An independent *t*-test or Fisher-Pitman Permutation test was used for analysis of normal distribution data, while a non-parametric Mann-Whitney *U*-test or Exact Mann-Whitney rank sum test was used for non-normal distribution data. To correct for multiple testing, q values were calculated using the Benjamini and Hochberg method. For the distribution of the biomarkers, Volcano plots of the log fold-change versus negative log (base 10) *P*-values from *t-*tests were used to display the differences between CLI patients and non-CLI controls. Binary logistic regression analysis was used to assess independent predictors of T2DM with CLI. Receiver operating characteristic curve (ROC) analysis was used to find the cut-off point of Siglec-5 for predicting CLI in T2DM patients. Correlations were analyzed using the Pearson method or nonparametric Spearman method. *P* < 0.05 was considered to indicate statistical significance.
